# Descriptive analysis and prognostic factors in cats with myeloma‐related disorders: A multicenter retrospective study of 50 cases

**DOI:** 10.1111/jvim.17051

**Published:** 2024-03-22

**Authors:** Lorris Lecot, Isabelle Desmas‐Bazelle, Sarah Benjamin, Pauline De Fornel, Frédérique Ponce, Matthew Kornya, Loïc Desquilbet, Claire Beaudu‐Lange, Catherine Ibisch, David Sayag, Ghita Benchekroun, Jérémy Béguin

**Affiliations:** ^1^ Ecole Nationale Vétérinaire d'Alfort–CHUVA Service de Médecine Interne Maisons‐Alfort France; ^2^ Université de Lyon, VetAgro Sup, Service de médecine interne Marcy l'Etoile France; ^3^ Royal Veterinary College London UK; ^4^ Davies Veterinary Specialists Hitchin UK; ^5^ Micen Vet Créteil France; ^6^ Université de Lyon, VetAgro Sup, Service de cancérologie, UR ICE Marcy l'Etoile France; ^7^ Ontario Veterinary College University of Guelph Guelph Ontario Canada; ^8^ Ecole nationale vétérinaire d'Alfort, IMRB Maisons‐Alfort France; ^9^ Clinique Vétérinaire de la Pierre bleue Pipriac France; ^10^ Nantes‐Atlantic College of Veterinary Medicine and Food Sciences (Oniris) Nantes France; ^11^ Onconseil Toulouse France; ^12^ Ecole Nationale Vétérinaire d'Alfort Université Paris Est Créteil, INSERM, IMRB Maisons‐Alfort France; ^13^ UMR1161 VIROLOGIE, INRAE, Ecole Nationale Vétérinaire d'Alfort, ANSES Université Paris‐Est Maisons‐Alfort France

**Keywords:** cat, oncology, plasma cell, prognosis

## Abstract

**Background:**

Myeloma‐related disorders (MRDs) are rare and poorly documented neoplasms of cats.

**Hypothesis/Objectives:**

To describe clinical, clinicopathologic, and imaging findings, response to treatment, and survival time and to identify factors associated with shorter outcomes in cats with MRD.

**Animals:**

Fifty cats with a diagnosis of MRD.

**Methods:**

Cats with paraproteinemia confirmed by serum protein electrophoresis (SPE) and either intramedullary plasmacytosis >10%, marked cytonuclear atypia with intramedullary plasmacytosis that ranged between 5% and 10%, or cytologically or histologically confirmed visceral infiltration were retrospectively included from several veterinary referral centers.

**Results:**

Bone marrow plasmacytosis and splenic or hepatic involvement were present in 17/27 cats (63%), 36/42 cats (86%), and 27/38 cats (71%), respectively. Anemia was reported in 33/49 cats (67%) and thrombocytopenia in 16/47 cats (34%). Some of the treatments that the cats received included melphalan and prednisolone (n = 19), cyclophosphamide and prednisolone (n = 10), chlorambucil and prednisolone (n = 4), prednisolone (n = 4), or other (n = 4). The overall response rates to melphalan, cyclophosphamide, and chlorambucil in combination with prednisolone were 87%, 90%, and 100%, respectively. Adverse events to melphalan or cyclophosphamide occurred in 65% and 23% of cats, respectively. Median survival time was 122 days (range, 0‐1403) and was not significantly associated with chemotherapy protocol. Anemia (hazard ratio [HR], 3.1; 95% confidence interval [CI], 1.0‐9.8) and thrombocytopenia (HR, 2.7; 95% CI, 1.2‐6.0) were risk factors for shorter survival.

**Conclusions and Clinical Importance:**

Our study confirmed the guarded prognosis of MRD in cats and identified risk factors for shorter survival times.

**Abbreviations**
AEs: adverse events
AGR: albumin‐to‐globulin ratio
BM: bone marrow
CI: confidence interval
CKD: chronic kidney disease
COP: cyclophosphamide, vincristine and prednisolone
CR: complete remission
CT: computed tomography
EMP: extramedullary plasmacytoma
HR: hazard ratio
HVS: hyperviscosity syndrome
LCOP: L‐asparaginase, cyclophosphamide, vincristine and prednisolone
MM: multiple myeloma
MRD: myeloma‐related disorders
MST: median survival time
NLR: neutrophil‐to‐lymphocyte ratio
PD: progressive disease
PLR: platelet‐to‐lymphocyte ratio
RI: reference interval
RR: response rate
SPE: serum protein electrophoresis

## 1 INTRODUCTION

Myeloma‐related disorders (MRD) represent <1% of all malignancies and 1.9% of hematologic malignancies in cats.^1^, ^2^ In dogs, the diagnostic criteria of multiple myeloma (MM) include the presence of >20% neoplastic plasma cells in the bone marrow (BM) and at least one the following criteria: serum paraproteinemia, immunoglobulin light chain (Bence Jones) proteinuria, and osteolytic lesions.^3^, ^4^, ^5^ Historically, the previous diagnostic criteria have been applied to cats.^6^, ^7^, ^8^ However, abdominal organ involvement without substantial BM infiltration appears more frequently in cats than in dogs.^9^, ^10^, ^11^ Indeed, splenic (83%‐100%) or hepatic (89%‐100%) infiltration and atypical plasma cell morphology (67%‐93.3%) without substantial BM infiltration were commonly reported in cats with MRD.^9^, ^10^, ^11^ The distinction between MM and multicentric noncutaneous extramedullary plasmacytoma (EMP) within the MRD is unclear because of the occurrence of systemic involvement in cats. Multiple myeloma (cases with BM plasmacytosis) and multicentric noncutaneous EMP (spleen or liver plasmacytosis or both without BM involvement) will be referred to as MRD in our study. Current recommendations require 2 of the following criteria for the diagnosis of MRD in cats: (1) >10% BM plasmacytosis or >5% atypical plasma cells; (2) paraproteinemia; (3) osteolytic lesions; (4) light chain proteinuria; and, (5) visceral organ involvement.^1^, ^10^

Only 4 studies on MRD in cats assessing treatment response with an alkylating agent in combination with prednisolone have been published.^8^, ^9^, ^10^, ^12^ One study reported a 71% response rate (RR) to melphalan and prednisolone.^12^ However, this protocol was associated with myelosuppression leading to treatment discontinuation.^12^ Good tolerance and a RR of 83% were reported with cyclophosphamide and prednisolone, which currently is considered the first‐line treatment for MRD in cats.^12^

Median survival time (MST) of cats diagnosed with MRD appears to be worse than for dogs, ranging between 5 days and 12.3 months.^8^, ^9^, ^10^, ^12^ Although prognostic factors have been identified in dogs, no prognostic factors have been established in cats.^13^, ^14^, ^15^, ^16^, ^17^, ^18^, ^19^, ^20^, ^21^, ^22^, ^23^, ^24^, ^25^, ^26^, ^27^, ^28^, ^29^, ^30^, ^31^, ^32^, ^33^, ^34^, ^35^ Classification into 2 categories (aggressive vs nonaggressive) based on several negative prognostic criteria (hypercalcemia, osteolytic lesions with pathological fractures, anemia, Bence Jones proteinuria, azotemia, persistently high serum protein concentration after 8 weeks of treatment and little or no clinical improvement) has been proposed for cats.^8^ Median survival time of 5 days for the aggressive category and 387 days for the less aggressive category were reported, but no rigorous statistical analysis was performed.^8^

Our primary objective was to describe the epidemiological and clinicopathological findings of systemic MRD in cats. The second objective was to describe outcomes and tolerance to treatment. The third objective was to identify factors associated with shorter survival times (ST).

## 2 MATERIALS AND METHODS

### 2.1 Case selection

The medical records of cats diagnosed with MRD from January 2005 to June 2022 at 14 referral centers were retrospectively reviewed (Royal Veterinary College, National Veterinary School of Alfort, Davies Veterinary Specialists, Micenvet, Ontario Veterinary College, VetAgro Sup, the Royal (Dick) School of Veterinary Studies, Onconseil, Oniris, Advetia, AURA veterinary, CENTREDMVET DMV, Liverpool University, Veterinary clinic of la Pierre Bleue).

### 2.2 Diagnostic criteria of MRD

Cats were retrospectively included based on the presence of hyperglobulinemia attributable to paraproteinemia as evidenced by serum protein electrophoresis (SPE) and 1 of the following criteria: BM plasmacytosis >10% or marked cytonuclear atypia if the BM plasmacytosis ranged between 5% and 10% or visceral plasma cell infiltration confirmed by cytological or histological examination. Medical records were reviewed by a single author (LL). No minimal duration of follow‐up was required.

### 2.3 Data collection

The following data were obtained from the medical records: breed, age, sex, weight, spay or neuter status, comorbidities and concomitant medications, clinical signs, and physical examination findings. The results of CBC and blood smear examination, biochemical analysis, serum (SPE) and urine protein electrophoresis and immunoglobulin type as determined by radial immunodiffusion, urinalysis, noninvasive blood pressure, fundic examination, thoracic and abdominal radiographs or ultrasound examinations, computed tomography (CT) scans, BM aspiration, cytological or histological analyses, and immunohistochemistry analyses were recorded. Regarding treatment, drug, dosage, frequency of administration, time to response (partial or complete), rescue treatment protocols and adverse events (AEs) were recorded. Adverse events associated with chemotherapy were graded according to the Veterinary Cooperative Oncology Group criteria for AEs.^36^ Regarding outcome, remission status, duration of remission, date of relapse (defined as occurrence of progressive disease [PD] after complete remission [CR] or partial response [PR]), date of last follow‐up if lost to follow‐up, date and cause of death and necropsy results if performed were recorded. Complete remission was defined as the resolution of hyperglobulinemia. Partial response was defined as at least a 30% decrease in globulin or the total protein concentrations without complete resolution, PD as at least a 20% increase in globulin or the total protein concentrations, and stable disease (SD) as an insufficient change to qualify for either PR or PD.^12^ Partial response, PD, and SD were defined based on total protein concentrations because serum globulin concentration was not measured at each assessment.^12^ The time to response was determined as the duration from the initiation of treatment to the occurrence of the first documented CR or PR. Follow‐up assessments were conducted every 10 to 15 days in the first month and subsequently at least once a month until CR, PD, or chemotherapy‐related toxicity was noticed. For patients achieving CR, reevaluation were performed every 2 months.

### 2.4 Statistical analysis

All statistical analyses were performed using commercially available software (SAS University Edition) under the supervision of an epidemiologist (LD). Continuous variables are presented as medians (range). Qualitative variables are presented as a percentage corresponding to the number of cats affected out of the number of cats for which the information was available. To identify factors associated with overall survival time (OST) and to compare outcomes between initial chemotherapy treatments (cyclophosphamide vs melphalan), survival analysis was performed by using Kaplan‐Meier curves and the log‐rank test. Survival time was defined as the time between diagnosis and either the date of all‐cause death or the date of censoring. Censored individuals were those either still alive at the end of the study (June 01, 2023) or those lost to follow‐up. Individuals were censored at the time of the last follow‐up available. The following exposures were used as dichotomous variables: anemia (PCV < 30%), azotemia (serum creatinine concentration > 1.58 mg/dL [140 μmol/L]), total (tCa) and ionized (iCa) hypercalcemia (tCa > 2.8 mmol/L or iCa > 1.4 mmol/L), osteolytic lesions, splenic or hepatic involvement or both (cytologically or histologically confirmed), increased liver enzyme activity (alkaline phosphatase or alanine aminotransferase), hyperbilirubinemia, hypoalbuminemia, marked hyperglobulinemia (defined below), low albumin‐to‐globulin ratio (AGR; defined below), proteinuria (urine protein‐to‐creatinine ratio > 0.4), Bence Jones proteinuria on urine protein electrophoresis, BM infiltration, BM cell atypia, rouleaux formation, monoclonal vs biclonal hypergammaglobulinemia on SPE, leukopenia, neutropenia, lymphopenia, thrombocytopenia, circulating plasma cells, high neutrophil‐to‐lymphocyte ratio (NLR; defined below), high platelet‐to‐lymphocyte ratio (PLR); (defined below), hyperviscosity syndrome (HVS), systemic arterial hypertension, pleural or abdominal effusion, positive FIV or FelV SNAP test (IDEXX Labs). As reference interval (RI) was similar among institutions for anemia, azotemia, hypercalcemia and proteinuria, clear cutoffs were selected for these variables. Increased liver enzyme activity, hyperbilirubinemia, hypoalbuminemia, leukopenia, neutropenia, lymphopenia and thrombocytopenia were defined as results outside of the individual laboratory RIs. Hyperviscosity syndrome was defined by the presence of at least 1 of the following: bleeding diathesis (epistaxis, petechiae, ecchymosis, gingival bleeding), central nervous system signs (seizures, stupor, coma), or fundus examination abnormality (dilated or tortuous retinal vessels, retinal hemorrhage, retinal detachment). No established cutoffs for AGR, NLR, PLR and hyperglobulinemia are available for cats diagnosed with MRD. Therefore, these variables were recoded into binary variables according to the first or the second tertile. A low AGR was defined as a value below the first tertile of the cohort. High NLR, high PLR, and marked hyperglobulinemia were defined as results above the second tertile of the cohort. Survival analysis was performed based on the aggressive vs nonaggressive classification.^8^

Crude and adjusted hazard ratios along with their 95% confidence intervals were calculated using Cox proportional hazards analysis. The variables included in the multivariate model as potential confounders were variables that were significant at *P* < .20 in the univariate analysis. Statistical significance was set at *P* < .05.

## 3 RESULTS

### 3.1 Patient signalment

Fifty cats (43 domestic shorthair cats, 22 Bengal cats, 2 Maine coon cats, 1 Norwegian cat, and 1 Chartreux cat) were included. Thirty‐two cats (64%) were castrated males, and 18 cats (36%) were spayed females. The median age was 12.0 years (range, 4.0‐17.5 years). The median body weight was 4.85 kg (range, 2.64‐9.90 kg).

### 3.2 Clinical and paraclinical findings

Presenting complaints, clinical signs, and paraclinical findings are summarized in Tables 1 and 2.

**TABLE 1:** Presenting complaints, clinical signs and comorbidities of cats diagnosed with MRD.

Observation	Number of cats affected/number of cats tested	Percentage of cats affected
Lethargy	34/50	68%
Anorexia	32/50	64%
Weight loss	31/50	62%
Pale mucous membrane	15/50	30%
Polyuria/polydipsia	14/50	28%
Dehydration	14/50	28%
Cardiac arrythmia/murmur	14/50	28%
Abdominal organomegaly	12/50	24%
Vomiting	11/50	22%
Ocular abnormalities^a^	5/26	19%
Periodontal disease	8/50	16%
Fever	7/50	14%
Recurrent respiratory infection	7/50	14%
Lameness	4/50	8%
Neurologic signs^b^	4/50	8%
Diarrhea	3/50	6%
Bleeding disorders	3/50	6%
Peripheral lymphadenopathy	3/50	6%
Cutaneous lesions	1/50	2%
Comorbidities		
Chronic kidney disease (treatment = renal diet)	2/50	4%
Hyperthyroidism (treatment = thiamazole)	2/50	4%
Feline lower urinary tract disease (treatment = urinary diet)	1/50	2%
Feline inflammatory bronchial disease (treatment = systemic prednisolone)	1/50	2%
Toxoplasmosis (treatment = clindamycine)	1/50	2%
Mycoplasmosis (treatment = doxycycline)	1/50	2%

^a^ Ocular abnormalities such as mydriasis, tortuous retinal vessels, retinal hemorrhage, retinal detachment or chorioretinitis.

^b^ Neurologic signs include abnormal mentation, seizure activity or cranial nerve deficits corresponding to thalamocortical or brainstem lesion.

**TABLE 2:** Paraclinical abnormalities of cats diagnosed with MRD.

Observation	Number of cats affected/number of cats tested	Percentage of cats affected	Median	Range
Diagnostic criteria				
Hyperglobulinemia^a^	50/50	100%	98 g/L	50–170 g/L
Hypergammaglobulinemia	44/44	100%		
Monoclonal	42/44	95%		
Biclonal	2/44	5%		
Bence Jones proteinuria	8/17	47%		
Bone marrow plasmacytosis	17/27	63%		
>10%	11/27	41%	30%	5%‐80%
<10%	6/27	22%		
Abdominal organ involvement^b^				
Spleen	36/42	86%		
Liver	27/38	71%		
Spleen and liver	23/37	62%		
Other	3/48 (kidney: 2 unilateral, 1 bilateral)	6%		
	1/48 (splenic and colic lymph nodes)	2%		
	1/48 (lung)	2%		
Thoracic organ involvement^b^	1/48 (mediastinal lymph node)	2%		
Osteolytic lesions^c^	11/38	29%		
Other paraclinical abnormalities				
Hypoalbuminemia^d^	29/49	59%	23 g/L	14‐37 g/L
Ionized hypercalcemia (iCa > 1.4 mmol/L)	6/36	17%	1.21 mmol/L	1.1‐1.65 mmol/L
Azotemia (creatinine >140 μmol/L)	20/48	42%	175 μmol/L	17‐1733 μmol/L
Increased liver enzyme activity^a^	16/40	40%	ALT: 61 U/L	ALT: 0‐1673 U/L
			ALP: 32 U/L	ALP: 6‐586 U/L
Hyperbilirubinemia^a^	5/27	19%	0.3 mg/dL	0‐19.6 mg/dL
Bone marrow plasma cell atypia	15/25	60%		
Anemia (PCV < 30%)	33/49	67%	22.1%	9.0%‐46.0%
Rouleaux formation	29/42	69%		
Leucopenia^d^	12/49	24%	6.2 × 10^9^/L	0.8‐26.0 × 10^9^/L
Neutropenia^d^	10/47	21%	4.7 × 10^9^/L	0.5‐24.5 × 10^9^/L
Lymphopenia^d^	27/47	57%	0.8 × 10^9^/L	0.1‐2.6 × 10^9^/L
Thrombocytopenia^d^	16/47	34%	156.5 × 10^9^/L	22.0‐650.0 × 10^9^/L
Circulating plasma cells	2/47	4%		
Hyperviscosity syndrome	9/27	33%		
Systemic arterial hypertension	3/17	18%	140 mmHg	84‐210 mmHg
Proteinuria (UPC > 0.4)	13/23	57%	1.0	0.3‐9.5
Pleural or abdominal effusion	12/49 (1 cat confirmed with bilateral myelomatous pleural effusion)	24%		
FIV/FeLV status^e^	1/8 (positive for FeLV antigen)	13%		

^a^ Above reference interval.

^b^ Visceral involvement based on ante‐mortem cytological or histological analyses.

^c^ Osteolytic lesions based on CT scan or radiographic examinations.

^d^ Below reference interval and confirmed by blood smear examination.

^e^ SNAP test (IDEXX Labs).

The most frequent presenting complaints were lethargy (68%), anorexia (64%), and weight loss (62%). At the time of diagnosis, all cats were hyperglobulinemic (median, 98 g/L; range, 50‐170 g/L). Biclonal gammopathy was recorded in 2/44 cats (5%). Thirty‐six of 42 cats (86%) had splenic involvement, and 27/38 (71%) had liver involvement. Twenty‐three of 37 cats (62%) had both splenic and hepatic involvement. Seventeen of 27 cats (63%) had BM plasmacytosis. Among them, 11/17 cats had BM plasmacytosis >10%, and 6/17 had BM plasmacytosis ranging between 5% and 10% with marked cytonuclear atypia. Ten of 23 cats (43%) had spleen or liver involvement without BM plasmacytosis. Two of 23 cats (9%) had BM plasmacytosis without spleen or liver involvement. Two of 47 cats had circulating plasma cells. Among these cats, 1 had BM plasmacytosis along with spleen and liver involvement and the other had spleen and liver involvement without BM plasmacytosis.

### 3.3 Diagnostic imaging

Imaging findings are summarized in Table 3.

**TABLE 3:** Imaging findings of cats diagnosed with MRD.

Observation	Number of cats affected/number of cats tested	Percentage of cats affected
Abdominal ultrasonography (n = 42)		
Splenic ultrasonographic abnormalities in cats with cytologic or histopathologic confirmed splenic involvement	22/36	61%
Splenomegaly	17/22	77%
Hyperechoic nodules	9/22	41%
Mottled appearance	8/22	36%
Hypoechoic nodules	4/22	18%
Hypoechoic parenchyma	4/22	18%
Hepatic ultrasonographic abnormalities in cats with cytologic or histopathologic confirmed hepatic involvement	24/27	89%
Hepatomegaly	13/24	54%
Hyperechoic parenchyma	7/24	29%
Hyperechoic nodules	5/24	21%
Mottled appearance	4/24	17%
Hypoechoic parenchyma	3/24	13%
Hypoechoic nodules	3/24	13%
Thoracic radiographs (n = 26)		
Normal	14/26	54%
Interstitial pulmonary pattern	5/26	19%
Pleural effusion	4/26	15%
Vertebral or costal osteolytic lesions	3/26	12%
Mediastinal lymphadenopathy	3/26	12%
Enlarged cardiac silhouette	3/26	12%
Lung nodule	1/26	4%
Localization of osteolytic lesions assessed by complete survey radiographs (n = 30) or whole body computed tomography (n = 8)		
Vertebral bodies	9/38	24%
Pelvis	4/38	11%
Spinous processes	3/38	8%
Ribs	2/38	5%
Humerus	2/38	5%
Femur	2/38	5%
Radius	1/38	3%
Ulna	1/38	3%
Scapula	1/38	3%
Phalanges	1/38	3%
Pathological fracture assessed by complete survey radiographs (n = 30) or whole body computed tomography (n = 8)	2/38	5%

Abdominal ultrasonography was performed at the time of diagnosis in 42 cats. For 3 cats, cytologic examinations of the liver and spleen were not consistent with visceral infiltration. Among these 3 cats, hepatomegaly was reported in 1 cat, whereas abdominal ultrasonography was within normal limits in the others.

In 2 cats, a bone biopsy was performed, and plasma cell infiltration of osteolytic lesions was confirmed. Pathological fractures were identified in 2 cats.

### 3.4 Treatment

Four cats were lost to follow‐up before treatment initiation. Treatment was initiated in 41/46 cats (89%). Nineteen cats (46%) received melphalan (0.1 mg/kg/day PO for 10 days and 0.05 mg/kg/day PO thereafter) and corticosteroids (prednisolone or dexamethasone) as initial chemotherapy. Ten cats (24%) were treated with cyclophosphamide (200 mg/m^2^ PO every 2‐3 weeks) and corticosteroids as first‐line treatment. Four cats (10%) received chlorambucil (2 mg per cat PO every other day) and corticosteroids as initial chemotherapy. Four cats (10%) were treated with corticosteroids alone as first‐line treatment. Four cats (10%) received other treatments, including dexamethasone and vincristine (n = 1), L‐asparaginase, cyclophosphamide, vincristine, and prednisolone (LCOP) protocol (n = 1), L‐asparaginase with corticosteroids and chlorambucil (n = 1) and L‐asparaginase with corticosteroids (n = 1) as initial chemotherapy. For all cats treated with prednisolone, the dosage ranged between 0.5 and 2 mg/kg/day.

Seven cats had several chemotherapy protocols either because of AEs or PD. Two cats had 4 protocols (chlorambucil/corticosteroid, followed by melphalan/corticosteroid, followed by cyclophosphamide/corticosteroid, followed by vincristine [n = 1]; cyclophosphamide/corticosteroid followed by melphalan/corticosteroid followed by lomustine, followed by cyclophosphamide/vincristine/prednisolone [COP] protocol [n = 1]). Two cats had 3 protocols (melphalan/corticosteroid, followed by cyclophosphamide/corticosteroid, followed by vincristine/corticosteroid [n = 1]; cyclophosphamide/corticosteroid, followed by chlorambucil/corticosteroid, followed by melphalan/corticosteroid [n = 1]). Three cats had 2 protocols (cyclophosphamide/corticosteroid, followed by melphalan/corticosteroid [n = 1]; melphalan/corticosteroid, followed by cyclophosphamide/corticosteroid [n = 1]; cyclophosphamide/corticosteroid, followed by chlorambucil/corticosteroid [n = 1]). The interval of drug administration was increased for 3 cats treated with melphalan and 1 with cyclophosphamide.

Analgesics such as gabapentin (8‐15 mg/kg PO q12h; n = 8), tramadol (2‐3 mg/kg PO q12h; n = 2) and alendronate (10 mg per cat PO once weekly; n = 1) also were administered.

### 3.5 Adverse events

Chemotherapy‐related AEs from both initial and rescue chemotherapy protocols are summarized in Table 4.

**TABLE 4:** Chemotherapy‐related adverse events in cats with MRD treated with prednisolone and melphalan, cyclophosphamide or chlorambucil.

Adverse events	Melphalan and prednisolone (n = 20)	Cyclophosphamide and prednisolone (n = 13)	Chlorambucil and prednisolone (n = 5)
**Neutropenia**	**11 (55%)**	**2 (15%)**	**0 (0%)**
Grade 1	3 (15%)	0 (0%)	0 (0%)
Grade 2	2 (10%)	2 (15%)	0 (0%)
Grade 3	3 (15%)	0 (0%)	0 (0%)
Grade 4	3 (15%)	0 (0%)	0 (0%)
Grade 5	0 (0%)	0 (0%)	0 (0%)
**Anemia**	**3 (15%)**	**1 (8%)**	**0 (0%)**
Grade 1	0 (0%)	0 (0%)	0 (0%)
Grade 2	1 (5%)	0 (0%)	0 (0%)
Grade 3	2 (10%)	1 (8%)	0 (0%)
Grade 4	0 (0%)	0 (0%)	0 (0%)
Grade 5	0 (0%)	0 (0%)	0 (0%)
**Thrombocytopenia**	**2 (10%)**	**0 (0%)**	**0 (0%)**
Grade 1	0 (0%)	0 (0%)	0 (0%)
Grade 2	1 (5%)	0 (0%)	0 (0%)
Grade 3	1 (5%)	0 (0%)	0 (0%)
Grade 4	0 (0%)	0 (0%)	0 (0%)
Grade 5	0 (0%)	0 (0%)	0 (0%)
**Gastrointestinal signs**	**2 (10%)**	**1 (8%)**	**0 (0%)**
Vomiting: grade 1	0 (0%)	1 (8%)	0 (0%)
Diarrhea: grade 2	1 (5%)	0 (0%)	0 (0%)
Nausea: grade 2	1 (5%)	0 (0%)	0 (0%)

*Note*: Grading according to VCOG criteria.^36^ Data are presented as number (%).

Among the cats for which follow‐up was available, AEs were reported in 13/20 cats (65%) treated with melphalan, 3/13 cats (23%) treated with cyclophosphamide and 0/5 cats treated with chlorambucil. No AEs were reported with other protocols.

### 3.6 Response

Among the 16 cats treated with melphalan and corticosteroids with evaluable follow‐up for response to treatment, 13 (81%) achieved CR, and 1 achieved PR (6%). The overall RR was 87%. The median time to response was 47 days (range, 14‐180 days). One cat achieved CR with melphalan after PD with chlorambucil. Among 10 cats treated with cyclophosphamide and corticosteroids with evaluable follow‐up, 7 cats (70%) achieved CR, and 3 cats achieved PR (30%). The overall RR was 100%. The median time to response was 27 days (range, 5‐161 days). One cat achieved CR with cyclophosphamide after PD with melphalan.

Among the 4 cats treated with chlorambucil and corticosteroids with evaluable follow‐up, 1 cat (25%) achieved CR, and 3 cats achieved PR (75%). The overall RR was 100%. The time to remission was only known in 3 cats and was 12 (PR), 30 (PR), and 42 (CR) days. One cat maintained PR after replacement of cyclophosphamide with chlorambucil.

Among the 4 cats treated with corticosteroids alone, only 1 of 3 evaluable cats experienced PR after 50 days of treatment. The overall RR was 33%. For the cats treated with dexamethasone and vincristine (n = 1), the LCOP protocol (n = 1), L‐asparaginase, chlorambucil and corticosteroids (n = 1) and L‐asparaginase with corticosteroids (n = 1) as initial chemotherapy, there was no assessment regarding the response to treatment.

### 3.7 Outcome

**FIGURE 1:**
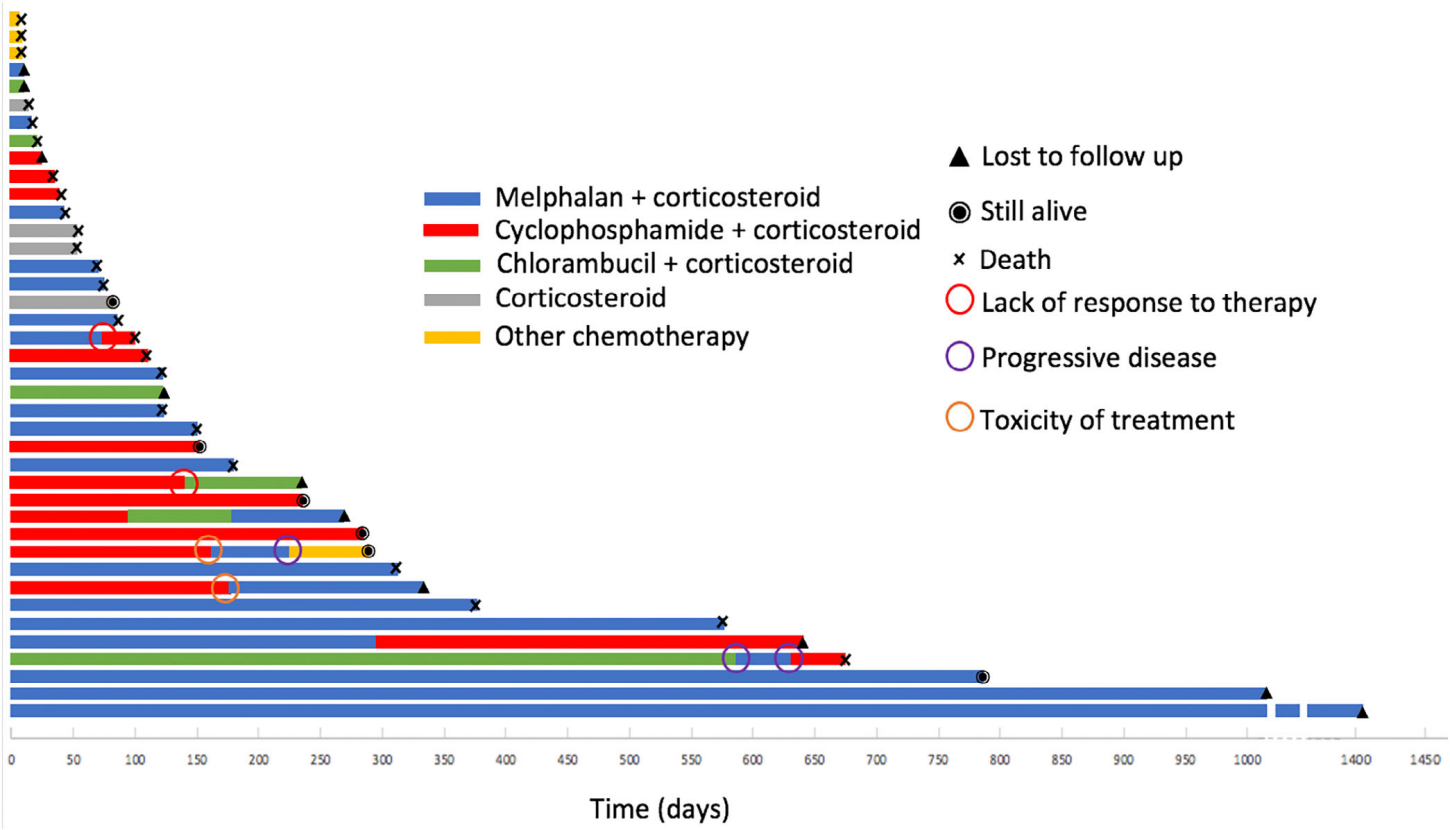
Outcome of treated cats (n = 41). One bar represents 1 cat. The abscissa represents time, and the ordinate represents each case.

The outcomes of the treated cats are summarized in Figure 1.

Fifteen cats were lost to follow‐up, and 6 were still alive at the end of the follow‐up period (153, 240, 284, 365, 780, and 1403 days after diagnosis). Twenty‐one cats were censored in the survival analysis. Of the remaining 29 cats, 27 cats were suspected to have died from MRD, 1 cat secondary to arterial thromboembolism, and 1 secondary to chronic kidney disease (CKD). The median follow‐up period was 80 days (range, 0‐1403 days).

### 3.8 Survival analysis

The estimated overall MST was 122 days. The MST of the cats treated with melphalan or cyclophosphamide plus corticosteroids as initial treatment, with the exclusion of cats not treated or treated with other protocols, was 315 days.

Median survival time was not significantly different between the cats treated with melphalan (145 days) or cyclophosphamide plus corticosteroids (median not reached) as initial chemotherapy (*P* = .30).

Results of CBCs were available for 40 cats for NLR analysis. The CBCs and blood smear examinations to evaluate platelet count were available for 38 cats for PLR analysis. Forty‐nine and 48 cats were included in the marked hyperglobulinemia and AGR analysis, respectively. For 1 cat, the globulin concentration was reported to be >120 g/L, and for the other cat, the albumin concentration was not available, precluding quantitative analysis and AGR measurement.

The cutoffs for AGR, NLR, PLR, and marked hyperglobulinemia as predictors of OST were 0.2 (range, 0.11‐0.64), 7.83 (range, 1.07‐93.33), 260 (range, 9.21‐844.16), and 110.7 g/L (range, 50.5‐170 g/L), respectively. Based on those cutoffs, the cats were divided into the following groups: high AGR (AGR ≥ 0.2; n = 29), low NLR (NLR < 7.83; n = 28), low PLR (PLR < 260; n = 26), and marked hyperglobulinemia (globulins ≥ 110.7 g/L; n = 15).

**FIGURE 2:**
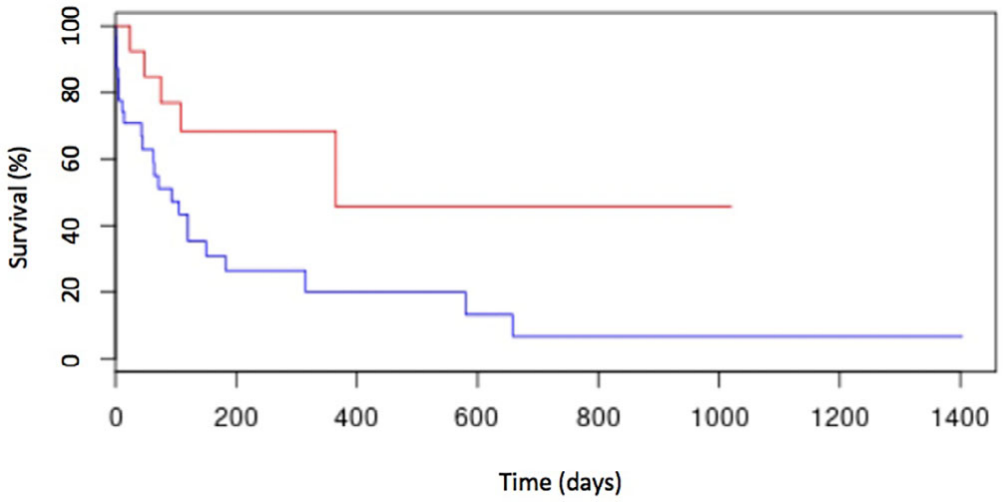
Kaplan–Meier curve of OST for cats with anemia (n = 32; blue line) and cats without anemia (n = 13; red line). Cats with anemia had an OST of 89 days (range, 0‐644 days), whereas cats without anemia had an OST of 356 days (range, 22‐356 days; *P* = .01).

**FIGURE 3:**
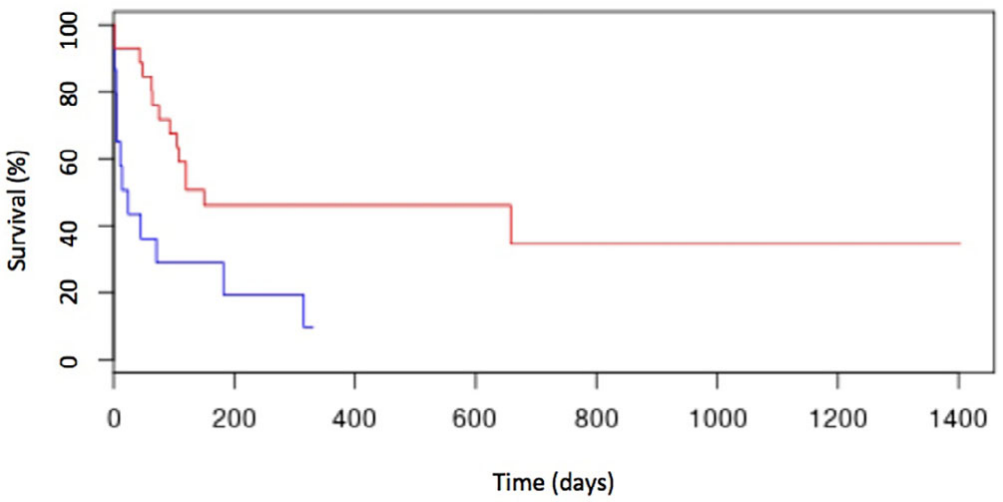
Kaplan–Meier curve of OST for cats with thrombocytopenia (n = 15; blue line) and cats without thrombocytopenia (n = 28; red line). Cats with thrombocytopenia had an OST of 22 days (range, 0‐311 days), whereas cats without thrombocytopenia had an OST of 156 days (range, 2‐656 days; *P* < .01).

**FIGURE 4:**
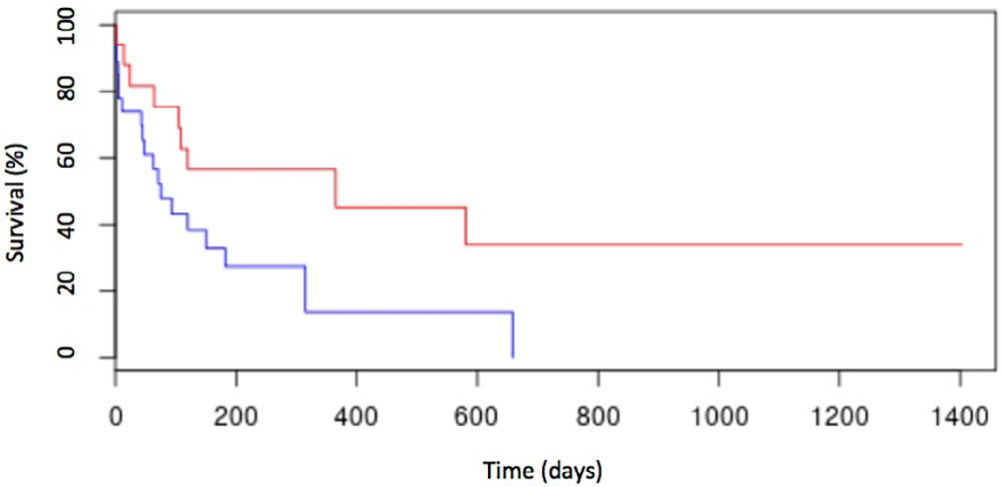
Kaplan–Meier curve of OST for cats with hypoalbuminemia (n = 27; blue line) and cats without hypoalbuminemia (n = 18; red line). Cats with hypoalbuminemia had an OST of 78 days (range, 0‐667 days), whereas cats without hypoalbuminemia had an OST of 367 days (range, 2‐589 days; *P* = .03).

**FIGURE 5:**
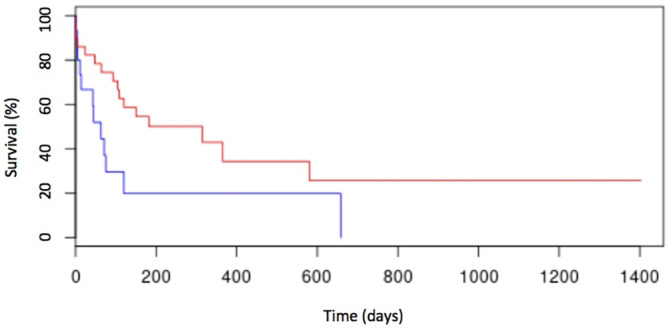
Kaplan–Meier curve of OST for cats with AGR ≤0.2 (n = 15; blue line) and cats with AGR > 0.2 (n = 29; red line). Cats with AGR ≤0.2 had an OST of 67 days (range, 2‐667 days), whereas cats with AGR > 0.2 had an OST of 322 days (range, 0‐589 days; *P* = .02).

In the univariate analysis, anemia (Figure 2), thrombocytopenia (Figure 3), hypoalbuminemia (Figure 4), and low AGR (Figure 5) at diagnosis were significantly associated with shorter ST. None of the other factors tested were significantly associated with shorter ST.

Variables with *P* < 0.2 in the univariate analysis (anemia, thrombocytopenia, hypoalbuminemia, and low AGR) were analyzed using Cox proportional hazards analysis. In the final multivariable model, anemia (*P* = .04) or thrombocytopenia (*P* = .02) at diagnosis were significantly associated with shorter ST (Table 5). Among cats with anemia, median hematocrit at diagnosis was 19.4% (range, 9%‐26.6%). Among cats with thrombocytopenia, median platelet count at diagnosis was 94 × 10^9^/L (range, 22‐138 × 10^9^/L).

**TABLE 5:** Results from the multivariate Cox proportional hazards model for all‐cause death in 50 cats with myeloma‐related disorders.

Variable	Hazard ratio	95% CI for HR		*P*‐value
		Lower	Upper	
Anemia (vs no anemia)	3.14	1.00	9.82	.04
Thrombocytopenia (vs no thrombocytopenia)	2.68	1.19	6.05	.02
Hypoalbuminemia (vs no hypoalbuminemia	1.91	0.78	4.69	.16
Low AGR (vs AGR ≥ 0.2)	0.96	0.34	2.74	.94

Abbreviations: CI, confidence intervals; HR, hazard ratio.

## 4 DISCUSSION

We report similar epidemiological and clinicopathological findings as previous case series.^6^, ^7^, ^8^, ^9^, ^10^, ^11^ Our study confirmed that visceral involvement was more common than BM plasmacytosis. Moreover, we confirmed good tolerance and RR associated with cyclophosphamide administration. In this cohort, anemia and thrombocytopenia were associated with shorter survival times, independent of hypoalbuminemia and low AGR.

Visceral organ involvement without BM infiltration appears frequent in cats with MRD.^9^, ^10^, ^11^ In cats with 10% to 20% BM plasmacytosis, diagnosis can be made on the assessment of plasma cell morphology in BM.^1^, ^9^, ^10^, ^11^ Normal BM contains <5% plasma cells.^1^ In our study, we included cases with BM plasmacytosis below 10% (n = 6) with cellular atypia and visceral organ involvement. Our study emphasizes visceral involvement and BM cellular atypia as main criteria for the diagnosis of MRD even if BM plasmacytosis is between 5% and 10%.^1^, ^9^, ^10^, ^11^

Our study shares several variables with prior studies. Indeed, our results emphasize the nonspecific nature of the clinical signs (lethargy, anorexia and weight loss) of MRD.^8^, ^10^, ^12^ As previously reported, males were overrepresented (64%) in our study.^8^, ^10^, ^12^ The sex predilection observed is similar in human medicine.^37^

The frequency of anemia, thrombocytopenia, azotemia, increase in liver enzyme activity, ionized hypercalcemia and Bence Jones proteinuria reported in our study was similar to previously studies.^8^, ^9^, ^10^, ^12^ Conversely, biclonal gammopathy was less frequent in our study (5%) compared with previous studies (20%‐25%).^8^, ^9^, ^10^ However, a recent study did not report any biclonal gammopathy on SPE in 10 cats.^12^ According to our results, it seems that biclonal gammopathy is infrequent. However, immunofixation electrophoresis would be required to confirm this finding.

Bone marrow plasmacytosis has been reported in 50% to 100% of cats with MRD.^8^, ^9^, ^10^, ^12^ In our study, BM plasmacytosis was diagnosed in 63% of cats, with one‐third of cats having BM plasmacytosis of <10%. These results emphasize that BM plasmacytosis is uncommon in cats with MRD and that infiltration may be mild in many cases.^1^, ^8^, ^10^, ^12^ In our study, 86% and 71% of cats had splenic and hepatic infiltration at diagnosis, respectively, confirming that visceral involvement is an important criterion for the diagnosis of MRD. Additionally, nonspecific ultrasonographic abnormalities were recorded in 61% and 89% of cats with confirmed splenic and hepatic infiltration, respectively. Thus, if MRD is suspected, splenic or hepatic ultrasound abnormalities or both are insufficient to establish a diagnosis. Cytology or histopathology or both are required to confirm involvement. In our study, normal abdominal ultrasonographic findings were recorded in 39% and 11% of cats with splenic and hepatic infiltration, respectively. This finding is consistent with previous studies in which a normal ultrasound examination could not rule out liver or spleen involvement.^8^, ^9^, ^10^, ^12^

Osteolytic lesions were identified in 29% of cases, which is lower than previous studies (50%‐66%).^8^, ^10^ Differences in imaging modalities among studies do not explain this result, because staging was performed mainly by skeletal radiographs in previous studies as in our study. Although fine needle aspirates of osteolytic lesions were not performed in our study, plasma cell infiltration was suspected in cats with consistent radiographic lesions. Pathologic fracture seems to be infrequent, because only 2 cats were affected in our study, and 1 of 40 cats in 3 previous studies.^8^, ^10^, ^12^ These results contrast with studies in humans, in which 80% of patients present with osteolytic lesions, osteoporosis or fractures on radiographs,^5^ and dogs, where 64% have osteolytic lesions and 2 of 38 dogs (5%) presented with pathologic fracture.^1^ The lower sensitivity of radiography compared with CT, positron emission tomography‐CT, and magnetic resonance imaging, which are used in human medicine, may decrease the sensitivity of lesion detection in dogs and cats.^5^, ^38^ As opposed to the previous study, osteolytic lesions were not associated with decreased MST in our study.^8^ Because CT and radiography were not routinely performed, osteolytic lesions may have been underdiagnosed, limiting rigorous statistical analysis.

In our cohort, the efficacy of melphalan or cyclophosphamide used in combination with prednisolone was similar to previously published data.^12^ Cats treated with melphalan or cyclophosphamide had RR of 88% and 91%, respectively. Survival times for cats initially treated with melphalan or cyclophosphamide were not significantly different. However, toxicity with the melphalan protocol was 3 times more frequent than with cyclophosphamide. These results were similar to a study where melphalan was more myelotoxic than cyclophosphamide.^12^ Although melphalan was associated with a high RR in our study, its common BM toxicity led to its temporary discontinuation in 3 cats (15%). However, as has been recently demonstrated in dogs with MM, a pulsed protocol of melphalan could be less toxic than a continuous protocol in cats.^13^ In our study, hematologic abnormalities after chemotherapy initiation were suspected to be secondary to treatment. Cytopenia secondary to PD was considered less likely based on normal globulin concentration. Conversely, cyclophosphamide was well tolerated, and only 2 cats developed grade 2 neutropenia, and 1 cat developed grade 3 anemia. These results combined with those of a previous study emphasize that the toxicity associated with cyclophosphamide is mostly transient and does not require alteration in treatment protocols.^12^ In our study, cats initially treated with chlorambucil and prednisolone had a satisfactory RR (100%) without toxicity. These results are similar to those of another study reporting mild and infrequent toxicity with chlorambucil.^12^ However, the small number of cats treated with chlorambucil precludes conclusions about the toxicity and efficacy of this protocol.

In humans, bortezomib is a proteasome inhibitor that serves as the first‐line drug for MM.^39^ Although it is not currently widely available in veterinary medicine, it has been used in a cat that had a favorable response.^40^ Additional studies evaluating toxicity and effectiveness in a larger cohort are needed.

In our study, fever and a history of recurrent respiratory signs were reported in 15% and 14% of cats, respectively. Fever was not reported at diagnosis of MRD in previous studies.^8^, ^9^, ^10^, ^12^ Respiratory signs have been reported in 6% to 20% of cases.^10^, ^12^ These findings may support concurrent immunosuppression. In our study, 1 cat was diagnosed with toxoplasmosis and another with mycoplasmosis at the time of diagnosis. Because these systemic infections often are associated with immunosuppression, we suspected that MRD may have more easily permitted development of infections. No cats developed an infection during chemotherapy. This finding is relevant because many cats treated with melphalan developed neutropenia. In contrast, other studies have described infections in cats during melphalan treatment, which led some authors to suggest the routine prescription of antibiotics.^8^, ^12^ The increased infection rate could be explained by the inhibition of normal plasma cell proliferation by neoplastic plasma cells and BM infiltration leading to leukopenia or chemotherapy‐related immunosuppression.^3^, ^8^, ^12^ In our study, only the 2 previously described cats received antibiotics. Because the risk of infection appeared to be low in our cohort, monitoring of cats with systemic MRD is recommended rather than systemic antibiotic prophylaxis.

Similar to other studies, the prognosis of cats with MRD in our cohort appears to be poor, with MST of 4 months.^8^, ^10^ This result contrasts with other data documenting MST of 8 to 13 months.^9^, ^12^ However, these prolonged ST were only reported in 2 studies where cats were treated with cyclophosphamide or melphalan with exclusion of survival analysis of the cats not treated at all or treated with other protocols.^9^, ^12^ In addition, our survival analysis also included intent‐to‐treat cats that were not treated for whatever reason. Those untreated cats could have decreased overall MST. When excluding cats that did not receive chemotherapy or were treated using protocols other than melphalan or cyclophosphamide in combination with prednisolone, the MST was 10.5 months in our cohort. Among cats with appropriate follow‐up until death, death was attributed to MRD‐related causes in 93% of cases. One cat died from arterial thromboembolism. Because no previous echocardiographic examination and no follow‐up until death were available, it is not possible to rigorously attribute the arterial thromboembolism to MRD (HVS or hypercoagulability associated with protein‐losing nephropathy), occult primary myocardiopathy or other diseases. Another cat died of kidney failure. The progression of CKD was suspected to be the cause of death because this cat was diagnosed with CKD for several years and did not develop ionized hypercalcemia, pyelonephritis, HVS, Bence Jones proteinuria, or nephromegaly consistent with plasma cell infiltration during follow‐up.

No prognostic factors associated with shorter ST have been established in cats diagnosed with MRD. One study grouped MRD into 2 categories (aggressive vs nonaggressive) based on criteria known to predict behavior in dogs.^8^ These results should be approached with caution because the cohort only consisted of 9 cats.^8^ Our study is the first to establish factors associated with shorter ST based on multivariate analysis in a large cohort. Because, at the time of writing, no established cutoffs for AGR, NLR, PLR, and hyperglobulinemia are available for cats diagnosed with MRD, tertile values were used as cutoffs to assess the association with some variables. Tertile values were preferred to quartile values to ensure a sufficient number of cases to obtain reliable results for statistical analysis. Tertile values were preferred to other methods (such as receiver operating curve analysis) to limit the influence of the second variable in determination of cutoffs. Because a low AGR could reflect more aggressive disease, the first tertile was chosen as a cutoff. High NLR^13^, ^27^, ^28^, ^29^, ^30^, ^31^, ^32^ was demonstrated to be a negative prognostic factor in humans and dogs with MM and high PLR^31^, ^32^ in humans. Therefore, the second tertile was chosen for NLR,^13^, ^27^, ^28^, ^29^, ^30^, ^31^, ^32^ PLR,^31^, ^32^ and hyperglobulinemia.

In a recent study, cats with MRD that were anemic at the time of diagnosis had a shorter MST (252 days) than nonanemic cats (289 days).^12^ The difference in MST between the 2 groups was not significant. In our study, anemia and thrombocytopenia were significantly and independently associated with shorter ST. Anemia and thrombocytopenia were defined according to the RI of the participating institutions. The RI for anemia (30%) was similar across all institutions. Because the RI for platelet count was different among institutions, individual laboratory RI was chosen. In 1 multicenter study, anemia was defined as a hematocrit <25%.^9^ Other non‐multicentric studies used cutoffs of 29.5%^12^ or 31.7%^10^ for anemia and 110 × 10^9^/L^12^ or 175 × 10^9^/L^10^ for thrombocytopenia. Despite the fact that thrombocytopenia was defined by different cutoffs in our study, it appears that the median and range of platelet count of thrombocytopenic cats were within the cutoffs defined by other studies. In previous studies, neither anemia nor thrombocytopenia were associated with worse outcome in dogs with MM.^1^, ^13^, ^14^ Conversely, anemia is also a recognized negative prognostic factor in humans with MM.^33^, ^34^, ^35^ The worse outcomes associated with anemia and thrombocytopenia could be related to severe BM infiltration, blood loss secondary to HVS or gastrointestinal bleeding or both. In our study, gastrointestinal bleeding was not recorded. Gastrointestinal bleeding was documented in 20% of dogs with MM.^41^ Recently, a case of MM involving the gastrointestinal tract was reported in a dog.^42^ Thus, cats with MRD and evidence of gastrointestinal bleeding may have gastrointestinal plasma cell infiltration. Anemia also may result from chronic illness, causing anemia of chronic disease and erythrophagocytosis.^43^, ^44^ Thrombocytopenia may result from viral diseases, disseminated intravascular coagulation, immune‐mediated thrombocytopenia, and thrombotic thrombocytopenic purpura.^45^, ^46^, ^47^, ^48^, ^49^ Considering the guarded prognosis associated with those diseases, it may worsen the prognosis.

In a previous study, cats presenting with hypoalbuminemia at diagnosis had a more than 3‐fold longer ST (684 days) than cats with normal albumin concentration (214 days).^12^ The risk of death for cats with normal albumin concentration vs hypoalbuminemia approached statistical significance, but a type II error may have affected the outcome.^12^ This result contrasts with our findings, which supported hypoalbuminemia and low AGR as variables significantly associated with shorter ST in the univariate analysis. This association was not significant after multivariate analysis, which may be a result of the small sample size. In dogs, neither hypoalbuminemia nor low AGR has been associated with shorter ST.^1^, ^13^, ^14^ Several studies have shown that hypoalbuminemia is a negative prognostic factor in people with MM.^50^, ^51^, ^52^ The specific cause of decreased serum albumin concentrations in MM is unknown. Some studies report that low serum albumin concentrations correlate with increased serum concentrations of interleukin‐6, a potent plasma cell growth factor reflecting disease severity and cell proliferation.^53^, ^54^, ^55^, ^56^, ^57^, ^58^, ^59^, ^60^, ^61^, ^62^, ^63^, ^64^ Low serum albumin concentrations also may be related to prolonged dysrexia.^65^ In human medicine, 1 study reported that patients with hypoalbuminemia were older and had lower hemoglobin concentrations, worse performance status, and higher concentrations of serum beta2‐microglobulin, serum M protein, and BM plasma cells.^50^ Because those clinical factors reflect disease severity in MM patients, hypoalbuminemia in cats also could be linked to a more severe disease course.

Our study had some limitations, mainly related to its multicenter retrospective nature. Despite being the largest study published, the population size is small and not homogeneous in terms of staging tests and modalities, initial treatment, follow‐up, and rescue therapy. Moreover, immunofixation electrophoresis was not performed at diagnosis, and the response to treatment was only based on serum globulin measurements. A recent study in dogs found that assessing response based on serum M‐protein concentration was associated with both specific clinical events and OST, whereas categorization based on serum globulin concentration was not.^66^, ^67^ These results led to the recommendation of the use of electrophoresis‐based techniques with evaluation of densitometric paraprotein as a means of monitoring animals with secretory MRD. Furthermore, osteolytic lesions and Bence Jones proteinuria were not reassessed during follow‐up. The variability in the reevaluation schedule could have led to calculation of a longer time to response than justified. Finally, the diagnosis of HVS only relied on clinical signs, and it would have been more reliable to perform measurements of blood viscosity and exclude other pathologies.

In conclusion, our findings suggest that MRD in cats manifests more frequently as hepatic or splenic infiltration than marked BM plasmacytosis and has a poor prognosis. Cyclophosphamide and corticosteroids as first‐line treatment appear to be well tolerated and, in our study, had no significant difference in MST compared to melphalan, although lack of a significant difference could have been related to small sample size. The estimated overall MST was 122 days. However, the MST of cats treated with melphalan or cyclophosphamide plus corticosteroids as initial treatment was 315 days.

Anemia and thrombocytopenia were found to be significantly and independently associated with shorter ST. Additional studies are warranted to validate the negative prognostic value of these markers in a larger number of cases.

## CONFLICT OF INTEREST DECLARATION

Authors declare no conflict of interest.

## OFF‐LABEL ANTIMICROBIAL DECLARATION

Authors declare no off‐label use of antimicrobials.

## INSTITUTIONAL ANIMAL CARE AND USE COMMITTEE (IACUC) OR OTHER APPROVAL DECLARATION

Approved by Comité d'éthique en Recherche Clinique (ComERC) Ecole nationale vétérinaire d'Alfort, number 2022‐10‐13 and by Veterinary Research Ethics Committee of Institute of Infection, Veterinary and Ecological Sciences, Leahurst Campus, Neston, South Wirral, reference VREC1265.

## HUMAN ETHICS APPROVAL DECLARATION

Authors declare human ethics approval was not needed for this study.
