# Erratum to “Descriptive analysis and prognostic factors in cats with myeloma‐related disorders: A multicenter retrospective study of 50 cases”

**DOI:** 10.1111/jvim.17274

**Published:** 2024-12-28

Lecot L, Desmas‐Bazelle I, Benjamin S, et al. Descriptive analysis and prognostic factors in cats with myeloma‐related disorders: A multicenter retrospective study of 50 cases. *J Vet Inter Med*. 2024; 38: 1693–1705. https://doi.org/10.1111/jvim.17051

In the above mentioned article, there is an error in section 3 Results, 3.1 Patient signalment, first sentence. The number of Bengal cats is 2 and not 22. The sentence should be as follows. “Fifty cats (43 domestic shorthair cats, 2 Bengal cats, 2 Maine coon cats, 1 Norwegian cat, 1 Siamese cat, and 1 Chartreux cat) were included.”

We apologize for this error.

